# Mobile Phone Ownership and Use Among Women Screening for Cervical Cancer in a Community-Based Setting in Western Kenya: Observational Study

**DOI:** 10.2196/28885

**Published:** 2022-06-07

**Authors:** Jacob Stocks, Saduma Ibrahim, Lawrence Park, Megan Huchko

**Affiliations:** 1 Center for Global Reproductive Health Duke Global Health Institute Durham, NC United States; 2 Kenya Medical Research Institute Nairobi Kenya; 3 Division of Infectious Diseases Department of Medicine Duke University School of Medicine Durham, NC United States; 4 Research Design and Analysis Core Duke Global Health Institute Durham, NC United States; 5 Department of Obstetrics and Gynecology Duke University School of Medicine Durham, NC United States

**Keywords:** cell phone, mobile health, mHealth, cervical cancer screening, Kenya, human papillomavirus, HPV testing

## Abstract

**Background:**

Mobile phone ownership among women of reproductive age in western Kenya is not well described, and our understanding of its link with care-seeking behaviors is nascent. Understanding access to and use of mobile phones among this population as well as willingness to participate in mobile health interventions are important in improving and more effectively implementing mobile health strategies.

**Objective:**

This study aims to describe patterns of mobile phone ownership and use among women attending cervical cancer screening and to identify key considerations for the use of SMS text message–guided linkage to treatment strategies and other programmatic implications for cervical cancer screening in Kenya.

**Methods:**

This analysis was nested within a cluster randomized trial evaluating various strategies for human papillomavirus (HPV)–based cervical cancer screening and prevention in a rural area in western Kenya between February and November 2018. A total of 3299 women were surveyed at the time of screening and treatment. Questionnaires included items detailing demographics, health history, prior care-seeking behaviors, and patterns of mobile phone ownership and use. We used bivariate and multivariable log-binomial regression to analyze associations between independent variables and treatment uptake among women testing positive for high-risk HPV.

**Results:**

Rates of mobile phone ownership (2351/3299, 71.26%) and reported daily use (2441/3299, 73.99%) were high among women. Most women (1953/3277, 59.59%) were comfortable receiving their screening results via SMS text messages, although the most commonly *preferred* method of notification was via phone calls. Higher levels of education (risk ratio 1.23, 95% CI 1.02-1.50), missing work to attend screening (risk ratio 1.29, 95% CI 1.10-1.52), and previous cervical cancer screening (risk ratio 1.27, 95% CI 1.05-1.55) were significantly associated with a higher risk of attending treatment after testing high-risk HPV–positive, although the rates of overall treatment uptake remained low (278/551, 50.5%) among this population. Those who shared a mobile phone with their partner or spouse were less likely to attend treatment than those who owned a phone (adjusted risk ratio 0.69, 95% CI 0.46-1.05). Treatment uptake did not vary significantly according to the type of notification method, which were SMS text message, phone call, or home visit.

**Conclusions:**

Although the rates of mobile phone ownership and use among women in western Kenya are high, we found that individual preferences for communication of messages about HPV results and treatment varied and that treatment rates were low across the entire cohort, with no difference by modality (SMS text message, phone call, or home visit). Therefore, although text-based results performed as well as phone calls and home visits, our findings highlight the need for more work to tailor communication about HPV results and support women as they navigate the follow-up process.

## Introduction

### Background

Cervical cancer disproportionately affects women in low- and middle-income countries (LMICs). It is the fourth most common cancer worldwide [1], with nearly 90% of cervical cancer–related deaths occurring in LMICs [1-3]. The vast majority of these deaths are preventable, as advances in screening methodologies, including cytology-based testing, have helped to decrease cervical cancer mortality in high-income countries [4,5]. Although the World Health Organization has recommended simplified and lower-cost screening strategies for LMICs, many countries such as Kenya still face a range of challenges in implementing and scaling cervical cancer prevention programs. These challenges include low numbers of trained health care providers, lack of physical and financial resources, complicated screening logistics, low community awareness of disease risk and screening opportunities, and personal health beliefs [6,7]. According to Kenya’s 2014 Demographic and Health Surveys (DHS), only 14% of women surveyed had ever been screened for cervical cancer; however, the country experiences the highest cervical cancer incidence rate within the East African region (33.8 per 100,000 women) and one of the highest in the world [1,8]. Thus, new and innovative approaches are required to overcome the current shortcomings in screening and linkage to treatment.

Human papillomavirus (HPV)–based screening, recently endorsed by the World Health Organization for use in screen-and-treat strategies in LMICs [9], can be collected via self-sampling, facilitating the decentralization of care from health facilities into community settings. In addition, HPV-based screening can be offered by nonphysician clinicians. This could increase the availability and acceptance of testing among women in low-resource settings. However, to be effective, HPV-based screening programs must be accompanied by effective counseling and education and electronic tracking systems for laboratory results and patient follow-up. One strategy to bridge these system-, provider-, and patient-level gaps is through the use of mobile phone technology. Mobile phone–based health (mobile health [mHealth]) interventions appear to be promising solutions to many of the infrastructure- and access-related challenges faced by LMICs and nonurban communities [10,11]. Many approaches to using telecommunications technology, including the collection of client data, medication adherence notifications, service reminders, and knowledge sharing campaigns, have been implemented in a variety of settings [12-15].

The efficacy of mHealth interventions relies, in part, on the level of access to and ownership of mobile phones among target populations. Improvements in cell phone network capability, decreasing costs of mobile phone ownership, and urbanization have led to growing mobile phone ownership throughout sub-Saharan Africa [16,17]. In recent decades, there has been a significant increase in mobile phone access in Kenya, with mobile subscription rates increasing from 0.02 to 86.1 per 100 people between 1997 and 2017 [16,18,19]. However, disparities in cell phone ownership and access by gender, residence type (urban or rural), educational attainment, and wealth remain [20]. Women constitute more than half of those currently unreached by the mobile phone market, and those who are poorer and less educated tend to be even less connected [21,22]. An analysis of mobile phone access from Kenya’s 2014 DHS revealed that 86.7% of women reported having a mobile phone in their household but did not provide insight into personal ownership, which is important to consider if potentially sensitive information is to be shared during an mHealth intervention [23]. Mobile phone ownership among women of reproductive age in western Kenya is not well described, and our understanding of its link with care-seeking behaviors is nascent. Understanding access to and use of mobile phones among this population as well as willingness to participate in mHealth interventions is important in improving and more effectively implementing mHealth strategies.

A recent cluster randomized trial in rural western Kenya showed that HPV testing via self-collection within community health campaigns (CHCs) was an acceptable and well-attended strategy for cervical cancer screening. However, the study found that treatment uptake among HPV-positive women was <50% [24]. Consequently, we used an enhanced strategy to link women to treatment, which used SMS text messages to provide women with screening results, educational content, and treatment reminders.

### Study Objectives

The objectives of this study were (1) to describe patterns of mobile phone ownership and use among women attending cervical cancer screening, (2) to identify key considerations for the use of SMS text message–guided linkage to treatment strategies and other programmatic implications for cervical cancer screening in Kenya, and (3) to determine whether mobile phone ownership or the method of results notification are independent predictors of treatment uptake among women who tested positive for HPV.

## Methods

### Study Setting and Sample

This analysis was nested within a cluster randomized trial evaluating various strategies for HPV-based cervical cancer screening and prevention in a rural area of western Kenya [24]. The cluster randomized trial enrolled women eligible for screening based on the Kenya Ministry of Health guidelines (aged 25-65 years with an intact uterus and cervix). High-risk human papillomavirus (hrHPV)–based screening was offered free of charge via self-sampling to women in health facilities or CHCs. To address levels of treatment uptake of <50%, the study staff engaged key stakeholders to develop an *enhanced linkage-to-treatment* strategy. The strategy included decentralized treatment sites with increased frequency and educational content via mobile phone messaging for women who had been screened for HPV. This study analyzed mobile phone ownership data and treatment uptake from the trial after the implementation of the enhanced strategy, which was identical across arms.

### Study Design

A total of 6 CHCs were conducted in Migori County, Kenya, between February and November 2018. Each CHC lasted 2 weeks and took place at different sites around the community with a predetermined schedule that was promoted during community mobilization. After self-collection and laboratory processing, the study staff notified women of their results via their preferred notification method: phone calls, SMS text messages, or home visits. Those who tested positive for hrHPV were referred to a treatment site deemed most accessible based on their community, where cryotherapy was available 5 days per week at no charge to the participants.

Data for this analysis originated from questionnaires administered at the time of screening and treatment (Multimedia Appendices 1 and 2). After informed consent was obtained, trained study staff verbally delivered the questionnaires and recorded participant responses electronically with tablets using OpenDataKit. The questionnaires included items detailing demographics, health history, prior care-seeking behaviors, and patterns of mobile phone ownership and use. Methods for data privacy and storage as well as specimen collection and storage have been described elsewhere [24].

### Quantitative Analysis

Women who attended one of the CHCs during the study period, consented to participate, completed the prescreening questionnaire, and were screened were included in this analysis. Basic descriptive statistics were used to characterize the study population. These included frequencies and percentages of demographic factors. In addition, we analyzed factors related to the implementation of SMS text messaging in screening and treatment strategies and their association with mobile phone ownership, compared using the chi-square test of independence. We considered women who reported personal ownership of their most frequently used mobile phone as mobile phone owners and those who did not report personal ownership but did report the use of a mobile phone as mobile phone sharers. Women who reported never having used a mobile phone were considered as nonusers. We carried out bivariate log-binomial regression to analyze associations between independent variables, including mobile phone ownership and chosen method of result notification, and the main outcome variable, attendance at treatment after having screened positive for hrHPV. We dichotomized treatment attendance, defined as attending a designated treatment facility within 3 months (not self-reported), as *no*=0 (did not attend treatment) and *yes*=1 (attended or attempted treatment). This distinction was made regardless of whether participants actually received treatment, as some were deemed ineligible for treatment because of pregnancy at the time of presentation, menses, or suspicion of cervical cancer. To control for potential confounding factors, age and all variables associated with the outcome (significant at the *P*<.10 level) in the bivariate analysis were included in the multivariable log-binomial regression analyses. We reported adjusted risk ratios and 95% CI resulting from multivariable log-binomial regression analysis and considered statistical significance at the 5% significance level (two-sided *P*<.05) for all tests. All aforementioned analyses were performed using STATA/SE 17 software (Stata Corporation).

### Ethics Approval

We obtained ethics approval for this study from the ethics review unit of the Kenya Medical Research Institute (protocol #2918) and the institutional review board of Duke University (protocol #Pro00077442). The parent cluster randomized trial was registered at ClinicalTrials.gov (NCT02124252). All participants provided written consent at the time of screening. The participants provided verbal affirmation during all follow-up visits.

## Results

Overall, 3299 women attended and were screened for HPV at one of the study CHCs. The average age was 38.2 (SD 11.3) years, with 60.02% (1980/3299) of the women aged between 25 and 39 years (Table 1). A large majority of the participants (2779/3299, 84.24%) reported having a primary school education or less, and very few participants (102/3299, 3.09%) had completed a collegiate degree. Nearly all women (3212/3299, 97.36%) who were screened were either married or widowed, and most (2364/2521, 93.77%) of those who reported having a partner lived with that person. The majority of women (1893/3299, 57.38%) worked outside of the home, and overall, women had an average of 4.9 (SD 2.9) children. Few women (497/3299, 15.06%) had previously been screened for cervical cancer, whereas almost all women (3185/3299, 96.54%) had previously been tested for HIV, and 24.29% (773/3182) of women self-reported that they were living with HIV. Although nearly all women (3252/3299, 98.58%) reported being sexually active, less than half of them (1328/3299, 40.25%) reported using modern family planning methods. The overall hrHPV positivity rate during CHCs was 16.70% (551/3299; Table 1).

**Table 1:** Demographic characteristics of the study population by mobile phone ownership in a prospective study of mobile phone ownership in Migori, Kenya, between February and November 2018.

Characteristic			Total (N=3299)		Owners (n=2351)		Sharers (n=394)		Nonusers (n=554)
Age (years), mean (SD)			38.2 (11.3)		38.0 (10.9)		35.4 (10.9)		40.5 (12.6)
**Age (years; n=3295), n (%)**									
	25-29	955 (29)		647 (27.5)		162 (41.1)		146 (26.5)	
	30-39	1025 (31.1)		765 (32.6)		112 (28.4)		148 (26.8)	
	40-49	686 (20.8)		520 (22.1)		67 (17.0)		99 (17.9)	
	50-59	453 (13.8)		312 (13.3)		39 (9.9)		102 (18.5)	
	60-65	176 (5.3)		105 (4.5)		14 (3.6)		57 (10.3)	
**Relationship status, n (%)**									
	Single	37 (1.1)		27 (1.1)		5 (1.3)		5 (0.9)	
	Single with partner	11 (0.3)		9 (0.4)		1 (0.2)		1 (0.2)	
	Married	2510 (76.1)		1779 (75.7)		348 (88.3)		383 (69.1)	
	Widowed	702 (21.3)		502 (21.3)		39 (9.9)		161 (29.1)	
	Separated or divorced	39 (1.2)		34 (1.5)		1 (0.3)		4 (0.7)	
**Live with partner (n=2521), n (%)**									
	Yes	2364 (93.8)		1655 (92.6)		341 (97.7)		368 (95.8)	
	No	157 (6.2)		133 (7.4)		8 (2.3)		16 (4.2)	
**Education level, n (%)**									
	Primary school or less	2779 (84.2)		1900 (80.8)		346 (87.8)		533 (96.2)	
	Some secondary school	520 (15.8)		451 (19.2)		48 (12.2)		21 (3.8)	
**Work outside of home, n (%)**									
	Yes	1893 (57.4)		1442 (61.3)		194 (49.2)		257 (46.4)	
	No	1406 (42.6)		909 (38.7)		200 (50.8)		297 (53.6)	
Number of children, mean (SD)			4.9 (2.9)		4.8 (2.8)		4.8 (3.1)		5.4 (3.1)
**Previous cervical cancer screening, n (%)**									
	Yes	497 (15.1)		413 (17.6)		41 (10.4)		43 (7.8)	
	No	2799 (84.8)		1937 (82.4)		353 (89.6)		509 (91.9)	
	Unsure	3 (0.1)		1 (0)		0 (0)		2 (0.3)	
**Previous testing for HIV, n (%)**									
	Yes	3185 (96.5)		2288 (97.3)		379 (96.2)		518 (93.5)	
	No	102 (3.1)		57 (2.4)		14 (3.6)		31 (5.6)	
	Unsure	12 (0.4)		6 (0.3)		1 (0.2)		5 (0.9)	
**HIV status (n=3182)^a^, n (%)**									
	Positive	773 (24.3)		587 (25.7)		63 (16.6)		123 (23.7)	
	Negative	2390 (75)		1688 (73.8)		313 (82.6)		389 (75.1)	
	Unsure	19 (0.6)		10 (0.4)		3 (0.8)		6 (1.2)	
**Currently using family planning or contraception, n (%)**									
	Yes	1328 (40.3)		996 (42.4)		165 (41.9)		167 (30.1)	
	No	1921 (58.2)		1323 (56.3)		221 (56.1)		377 (68.1)	
	Unsure	3 (0.1)		2 (0.1)		0 (0)		1 (0.2)	
	Not sexually active	47 (1.4)		30 (1.3)		8 (2)		9 (1.6)	
**Human papillomavirus result, n (%)**									
	Positive	551 (16.7)		393 (16.7)		66 (16.8)		92 (16.6)	
	Negative	2748 (83.3)		1958 (83.3)		328 (83.2)		462 (83.4)	

^a^A total of 3 participants refused to answer.

Among the 83.21% (2745/3299) of participants who reported having ever used a mobile phone, 85.64% (2351/2745) reported owning a mobile phone, and 14.31% (394/2745) reported sharing a mobile phone with their partner, child, family members, friends, neighbors, or other individuals. Compared with those who shared or did not use a mobile phone, mobile phone owners tended to be more highly educated, more commonly did not live with their partner, worked outside of the home at a greater proportion, and had fewer children. In addition, these women demonstrated greater health seeking behavior, as they had previously screened for HPV, tested for HIV, and used modern family planning methods at a higher proportion than mobile phone sharers and nonusers. Proportions of previous cervical cancer screening, HIV testing, and contraceptive use were the lowest among women who had never used a mobile phone. Self-reported HIV-positive status was more common among those who owned a mobile phone as compared with those who did not. No appreciable difference in hrHPV positivity was observed according to mobile phone ownership (Table 1).

Nearly three-quarters of women who shared their mobile phones did so with a spouse or partner, whereas few shared with children, other family members, friends, and neighbors. Although most women who reported having used a mobile phone said they used the device 7 days a week, a greater proportion of mobile phone owners used their device daily when compared with sharers (Table 2). Frequent technical issues were reported by both owners and sharers. A total of 56.81% (1559/2744) of women reported encountering challenges with use on a weekly basis, whereas just over approximately 13% (350/2744) reported daily issues. Approximately 15% (412/2744) of the participants stated that they never faced challenges using their device. Nearly two-thirds of women felt comfortable reading and receiving SMS text messages, whereas a quarter said they were unable to do so. Similarly, the majority of women were comfortable writing and sending SMS text messages. As expected, comfort with SMS text messages was not commonly reported by nonusers.

Most women (1953/3277, 59.60%) said that they would be comfortable receiving hrHPV test results via SMS text message, with 20.48% (671/3277) being very comfortable and 39.12% (1282/3277) being comfortable. Mobile phone owners and sharers dominated this majority, as only approximately 14% of nonusers reported comfort with SMS text message for notification of results (Table 3). However, when given the choice between different notification types, only 1 out of 4 women (25.98%) said that they would *prefer* to receive their results by SMS text message if negative, with 22.52% (743/3299) of women preferring SMS text message if their HPV result was positive. Of the 3.46% (114/3299) of participants whose preference for SMS text message changed based on possible HPV results, the overwhelming majority preferred a phone call for their results, whereas very few indicated a preference for home visits if their HPV result was positive. SMS text message was the only notification method that showed such variation based on potential screening outcome (Table 3). Preference for home visit result notification remained the same regardless of the hypothetical HPV result, whereas the proportion of women preferring a phone call decreased slightly if the result was positive. Regardless of mobile phone ownership, most women willing to receive results via SMS text message preferred notifications in Dholuo or English.

Overall, half of the surveyed women *preferred* to receive a phone call for results, either positive or negative, with the second most common method being home visits if positive and SMS if negative. However, notification method preferences varied significantly by mobile phone ownership. Nearly all women who reported personal ownership of a mobile phone preferred a phone-based method for notification of results (2100/2351, 89.32% if hrHPV-positive, and 2171/2351, 92.34% if hrHPV-negative). A lower proportion of mobile phone sharers preferred a phone-based notification (243/394, 61.7% if hrHPV-positive, and 251/394, 63.7% if hrHPV-negative). Unsurprisingly, very few nonusers preferred a phone-based notification (68/554, 12.3% if hrHPV-positive, and 71/554, 12.8% if hrHPV-negative; *P*<.001 if negative or positive).

In bivariable analysis, at least a secondary education, having missed work to attend screening, and previous cervical cancer screening resulted in a significantly higher risk of treatment uptake (crude risk ratio 1.23, 95% CI 1.02-1.50; crude risk ratio 1.29, 95% CI 1.10-1.52; and crude risk ratio 1.27, 95% CI 1.05-1.55, respectively; Table 4). In addition, the unadjusted risk of treatment uptake among women who shared a mobile phone with their spouse or partner was significantly lower than that among those who owned their own phone (crude risk ratio 0.65, 95% CI 0.43-0.97; Table 5). The unadjusted risk of treatment uptake was highest among those who received an SMS text message for result notification, although not significantly higher than phone calls or home visits in the bivariable analysis (Table 6). Number of children, working outside of the home, use of modern family planning methods, being told to attend screening by a family member, and frequency of mobile phone use were not significantly associated with treatment uptake, and therefore, they were not considered in multivariable analysis (Table 4). In multivariable analysis, the risk of treatment uptake for women who shared a mobile phone with their spouse or partner was lower than that for those who owned a mobile phone (adjusted risk ratio 0.69, 95% CI 0.46-1.05); however, the difference was not statistically significant. There was no appreciable difference in the risk of treatment uptake between mobile phone owners and nonusers (Table 5). In addition, when accounting for at least a secondary education, having missed work to attend screening, previous cervical cancer screening, and mobile phone ownership, the risk of treatment uptake did not vary significantly by notification type.

**Table 2:** Patterns of mobile phone ownership and use among women attending community-based cervical cancer screening in a prospective study in Migori, Kenya, between February and November 2018.

Technology use characteristics			Total (N=3299), n (%)		Owners (n=2351), n (%)		Sharers (n=394), n (%)
**Owner of commonly used mobile phone (n=2745)**							
	My own	2351 (85.6)		2351 (100)		N/A^a^	
	Spouse or partner	288 (10.5)		N/A		288 (73.1)	
	Child	34 (1.2)		N/A		34 (8.6)	
	Other family	30 (1.1)		N/A		30 (7.6)	
	Others	42 (1.5)		N/A		42 (10.7)	
**Frequency of mobile phone use among users (n=2745)**							
	<7 days a week	304 (11.1)		138 (5.9)		166 (42.1)	
	7 days a week	2441 (88.9)		2213 (94.1)		228 (57.9)	
**Frequency of technical issues (n=2744)^b^**							
	Never	393 (14.3)		346 (14.7)		47 (11.9)	
	At least once per month	313 (11.4)		274 (11.6)		39 (9.9)	
	At least once per week	1559 (56.8)		1379 (58.7)		180 (45.7)	
	At least once per day	350 (12.8)		305 (13.0)		45 (11.4)	
	Unsure	115 (4.2)		35 (1.5)		80 (20.3)	
	Other	15 (0.5)		11 (0.5)		3 (0.8)	
**Comfort reading and receiving SMS text message**							
	Not able	814 (24.7)		384 (16.3)		87 (22.1)	
	Very uncomfortable	68 (2.1)		39 (1.7)		7 (1.8)	
	Uncomfortable	295 (8.9)		149 (6.3)		45 (11.4)	
	Comfortable	1453 (44.0)		1197 (50.9)		190 (48.2)	
	Very comfortable	650 (19.7)		580 (24.7)		57 (14.5)	
	Unsure	19 (0.6)		2 (0.1)		8 (2.0)	
**Comfort writing and sending SMS text message (N=3298)^b^**							
	Not able	912 (27.6)		465 (19.8)		95 (24.1)	
	Very uncomfortable	68 (2.1)		39 (1.7)		6 (1.5)	
	Uncomfortable	372 (11.3)		215 (9.2)		54 (13.7)	
	Comfortable	1275 (38.7)		1046 (44.5)		176 (44.7)	
	Very comfortable	644 (19.5)		576 (24.5)		55 (14.0)	
	Unsure	27 (0.8)		10 (0.4)		7 (1.8)	

^a^N/A: not applicable.

^b^One participant refused to answer.

**Table 3:** Considerations for programmatic implementation and an SMS text message–guided linkage to treatment strategy based on a study of mobile phone ownership in Migori, Kenya, between February and November 2018.

Programmatic considerations		Total (N=3299), n (%)	Owners (n=2351), n (%)	Sharers (n=394), n (%)	Nonusers (n=554), n (%)
**Comfort receiving screening results via SMS text message (n=3277)^a^**					
	Very uncomfortable	327 (10)	153 (6.5)	25 (6.3)	149 (27.9)
	Uncomfortable	892 (27.2)	508 (21.6)	133 (33.8)	251 (47)
	Comfortable	1282 (39.1)	1058 (45)	165 (41.2)	59 (11)
	Very comfortable	671 (20.5)	604 (25.7)	55 (14)	12 (2.2)
	Unsure	105 (3.2)	27 (1.2)	15 (3.8)	63 (11.8)
**Preferred notification method if HPV^b^-negative^c^**					
	SMS text message	857 (26)	777 (33)	61 (15.5)	19 (3.4)
	Phone call	1636 (49.6)	1394 (59.3)	190 (48.2)	52 (9.4)
	Home visit	806 (24.4)	180 (7.7)	143 (36.3)	483 (87.2)
**Preferred notification method if HPV-positive^c^**					
	SMS text message	743 (22.5)	666 (28.3)	59 (15)	18 (3.2)
	Phone call	1668 (50.6)	1434 (61)	184 (46.7)	50 (9)
	Home visit	888 (26.9)	251 (10.7)	151 (38.3)	486 (87.7)
**Preferred language of SMS notification (n=897)**					
	English	184 (20.5)	174 (21.5)	7 (10.4)	3 (15)
	Kiswahili	164 (18.3)	142 (17.5)	20 (29.9)	2 (10)
	Dholuo	549 (61.2)	494 (61)	40 (59.7)	15 (75)

^a^A total of 22 participants refused to answer (20 of which were nonusers).

^b^HPV: human papillomavirus.

^c^Participants were asked about notification preferences at the time of screening, before knowing their HPV status. These are intended to convey women’s preferences in the event of a positive or negative result. This is not a comparison of method preference based on actual screening results.

**Table 4:** Factors associated with treatment uptake in bivariate analysis among women in a prospective study in Migori, Kenya, between February and November 2018 (n=551).

Characteristic			Treatment uptake		No treatment uptake		Crude risk ratio (95% CI)
Age (years), mean (SD)			36.4 (10.6)		35.5 (10.6)		1.00 (1.00-1.01)
**Education level, n (%)**							
	Primary school or less	224 (48.6)		237 (51.4)		—^a^	
	Some secondary school	54 (60)		36 (40)		1.23 (1.02-1.50)	
Number of children, mean (SD)			4.5 (2.8)		4.1 (2.7)		1.02 (1.00-1.05)
**Work outside of home, n (%)**							
	No	110 (46.6)		126 (53.4)		—	
	Yes	168 (53.3)		147 (46.7)		1.14 (0.96-1.36)	
**Missed work to attend screening, n (%)**							
	No	165 (45.8)		195 (54.2)		—	
	Yes	113 (59.2)		78 (40.8)		1.29 (1.10-1.52)	
**Told by family to attend screening, n (%)**							
	No	96 (46.2)		112 (53.8)		—	
	Yes	182 (53.1)		161 (46.9)		1.15 (0.96-1.37)	
**Previous cervical cancer screening, n (%)**							
	No	228 (48.5)		242 (51.5)		—	
	Yes	50 (61.7)		31 (38.3)		1.27 (1.05-1.55)	
**Currently using family planning or contraception, n (%)**							
	No	151 (49.5)		154 (50.5)		—	
	Yes	124 (52.5)		112 (47.5)		1.06 (0.90-1.25)	
	Not sexually active	2 (22.2)		7 (77.8)		0.45 (0.13-1.53)	
**Frequency of mobile phone use, n (%)**							
	<7 days a week	18 (38.3)		29 (61.7)		—	
	7 days a week	212 (51.5)		200 (48.5)		1.34 (0.92-1.95)	

^a^Reference category.

**Table 5:** Effect of mobile phone ownership on treatment uptake among women in Migori, Kenya, between February and November 2018 (n=551).

Mobile phone ownership			Treatment uptake, n (%)	No treatment uptake, n (%)		Crude risk ratio (95% CI)		Adjusted risk ratio^a^ (95% CI)	
Owners			207 (52.7)	186 (47.3)		—^b^		—	
**Sharers**									
	Spouse or partner	16 (34)		31 (66)	0.65 (0.43-0.97)		0.69 (0.46-1.05)		
	Other^c^	7 (36.8)		12 (63.2)	0.70 (0.39-1.27)		0.69 (0.38-1.25)		
Nonusers			48 (52.2)	44 (47.8)		0.99 (0.80-1.23)		1.06 (0.84-1.32)	

^a^Adjusted for having missed work to attend screening, prior cervical cancer screening, age, and education.

^b^Reference category.

^c^Includes children, other family, friends, neighbors, and others.

**Table 6:** Effect of notification type on treatment uptake among women in Migori, Kenya, between February and November 2018 (n=551).

Notification type	Treatment uptake, n (%)	No treatment uptake, n (%)	Crude risk ratio (95% CI)	Adjusted risk ratio^a^ (95% CI)
SMS text message	72 (56.3)	56 (43.7)	—^b^	—
Phone call	135 (48.7)	142 (51.3)	0.87 (0.71-1.05)	0.92 (0.76-1.12)
Home visit	71 (48.6)	75 (51.4)	0.86 (0.69-1.08)	0.89 (0.67-1.19)

^a^Adjusted for having missed work to attend screening, prior cervical cancer screening, age, education, and mobile phone ownership (owner, sharer, and nonuser).

^b^Reference category.

## Discussion

### Principal Findings

This study examined patterns of mobile phone ownership and use among women screening for cervical cancer in western Kenya. Mobile phone ownership rates and reported daily use were high, with more than three-quarters of women having ever used a mobile phone and ≥7 in 10 women owning their own phone. Most women were comfortable receiving their screening results via SMS text messages, although the most commonly *preferred* method of notification was via phone calls. Those who shared a mobile phone with their spouse or partner were less likely to attend treatment than those who owned a phone; however, overall, the method by which women received their screening results did not significantly impact their treatment uptake.

Understanding mobile phone ownership and comfort with use are essential for planning mHealth interventions. We observed rates of mobile phone ownership and use consistent with similar studies of women of reproductive age in sub-Saharan Africa, namely, Ethiopia, Burkina Faso, and Nigeria, which reported rates between 46% and 77% [25-27]. Although a recent study from northern Kenya reported mobile phone access at 99% among a small sample of women (n=104), our results seem more aligned with an analysis of mobile phone access conducted by Lee et al [23], who analyzed Kenya’s 2014 DHS mobile phone data in relation to contraceptive knowledge and use [28]. This nationally representative survey of 31,059 women reported mobile phone access of 87%, which is consistent with our data [23]. However, women surveyed for the DHS were asked about household-level access rather than personal-level ownership [23]. Such a distinction and related differences in women’s access are important to consider, especially given the sensitive nature of information that may be shared regarding HPV test results, treatment plans, and posttreatment instructions around sexual activity. Women who share a mobile phone with a partner or child may be less willing to engage in such interventions because of fear of unwanted disclosure or breach of privacy.

Many studies have identified links between mobile phone access and care-seeking behaviors [23,26,27,29]. Although the results of this study did not show a relationship between mobile phone ownership and treatment uptake, our data highlight many important considerations for the use of mHealth interventions. First, the ability to read, write, send, and receive SMS text messages among one’s target population is important to consider when designing text-based mHealth interventions. If populations have high levels of comfort with such tasks, as demonstrated by this survey, additional avenues of communication may be available between clients and providers. In addition to the unidirectional transmission of information, such as treatment or medication reminders, two-way communication is made possible, which could increase communication between client and provider and potentially reduce unnecessary visits to health facilities, freeing up time and space for health care workers. Such channels of communication can be maintained by live health workers or by automated chatbots and other algorithms. Given the high reported comfort with screening results via SMS text message, as well as comfort with reading, writing, sending, and receiving SMS text messages, such strategies appear feasible within western Kenya. Second, although mHealth has shown promise in bridging logistical gaps in similar situations, programs must ensure alternative means of communication or contact between health facilities and target populations to provide adequate and equitable access to women with varying levels of mobile connectivity or mobile phone access. In their work in Burkina Faso, Greenleaf et al [26] refer to such variance in access as “selective ownership” and argue that this can create an “ownership bias” in mHealth intervention uptake, making it difficult to reach populations most as risk. Such pitfalls can decrease intervention efficacy and alienate women who do not own or have access to a mobile phone, which based on our data, would exclude women with lower levels of educational attainment and poorer health seeking behaviors, putting them at risk for worse health outcomes. “Pre-intervention assessment,” as suggested by Jennings et al [27] conducting research in Nigeria, could illuminate such issues and allow health officials to preempt and address problems of equity for women in the intervention setting.

In addition to the aforementioned mHealth intervention considerations, this study illustrated the need for further research on a variety of topics related to the implementation of mHealth for cervical cancer screening. More information is needed to understand the lack of preference for text notification and why preferences change depending on the hrHPV status. The decrease in preference for SMS text message result notification if a participant was to screen hrHPV-positive compared with hrHPV-negative is likely a result of a desire for increased privacy, which could be related to cultural factors such as stigma. However, our data do not allow us to draw concrete conclusions on this, as we did not ask about factors that influenced preference, or lack thereof. Further studies should be conducted to survey women of reproductive age to help better understand these barriers and facilitators of SMS text message use and privacy as well as strategies for messaging at the time of screening, which would make SMS text messages more appealing. Second, frequent challenges and technical difficulties when using mobile phones were reported among the study population. These challenges could limit the feasibility and efficacy of an SMS text message–based system, as testing results and treatment reminders may be missed or not received owing to technical issues. Further examination of the nature of technical challenges and how they might impact the receipt of SMS text messages from program implementors is needed to further tailor this campaign. Third, regardless of the differences in treatment access by mobile phone ownership, the overall uptake remained low, even after the implementation of the enhanced linkage to the treatment strategy. Although this low level of treatment uptake (50.5%) improves upon treatment rates observed before implementation of the enhanced linkage strategy (between 31% and 39% uptake), the consistently low levels highlight the need for further exploration of opportunities for multipronged approaches to increasing uptake and access to care [24]. Identifying and bridging gaps in the cervical cancer prevention cascade is necessary to address the inequitable and preventable deaths caused by this disease. Finally, given the potential health benefits and increases in autonomy, there is a need to support increases in access to mobile phones and mobile phone networks for women of reproductive age [22].

### Limitations

Although we achieved a large sample size among the target population of women in rural Kenya, the study had a number of limitations. First, although treatment uptake was measured at the time of presentation to the clinic, the self-reported nature of our survey data limited our ability to make strong claims about the observed patterns of mobile phone ownership and use and how they impacted uptake. In addition, social desirability bias could have led to measurement errors, with women not accurately reporting health behaviors or mobile phone use given societal expectations. In addition, as services confirming receipt or review of SMS text messages would have imparted costs to the participants, we did not collect this information and were not able to report on how this may have affected treatment uptake. Although socioeconomic status was relatively homogenous among our study population, there was no strong operationalization of socioeconomic status within the survey. Therefore, it is difficult to conclude whether the observed associations are because of mobile phone ownership or a more upstream effect of economic status. Finally, our data may lack generalizability as we only considered women who attended a CHC, which is a self-selective action and could be affected by many factors. If such factors systematically inhibit a significant portion of women from these locations, the results of this study would be biased and not generalizable to the target population as a whole. A random, community-based, representative survey is warranted to evaluate the validity and generalizability of the findings of this study.

### Conclusions

This study examined the rates of mobile phone ownership, access, and the patterns of daily use among women of reproductive age in western Kenya. In addition, we highlighted many key considerations for the implementation of mHealth interventions in resource-limited settings, specifically those using SMS text messaging. Although rates of mobile phone ownership and use among women in western Kenya are high, we found that individual preferences for communication of messages about HPV results and treatment varied, and treatment rates were low across the entire cohort, with no difference by modality (SMS text message, phone call, or home visit). Further work is needed to tailor communication about HPV results and to support women as they navigate the follow-up process.

## Appendix

Multimedia Appendix 1 Questionnaire administered at screening.

Multimedia Appendix 2 Questionnaire administered at treatment.

## Abbreviations

CHC: community health campaign
DHS: Demographic and Health Surveys
HPV: human papillomavirus
hrHPV: high-risk human papillomavirus
LMIC: low- and middle-income country
mHealth: mobile health
