# Supplementary material for: Mobile Phone Ownership and Use Among Women Screening for Cervical Cancer in a Community-Based Setting in Western Kenya: Observational Study
Source: JMIR Public Health Surveill. 2022 Jun 7;8(6):e28885. doi: 10.2196/28885 (PMC9214615; doi:10.2196/28885)
Supplement: Multimedia Appendix 1 [file publichealth_v8i6e28885_app1.doc]

| **Multimedia Appendix 1**. Questionnaire administered at screening.  **CHC PRE AND POST HPV TEST INFORMATION SHEET** |
| --- |
| **Date of Testing (ODK will capture date of testing)**   1. Name of the staff administering the form (**dropdown**): *___________________*____*____________* |
| **PARTICIPANT PRE-HPV TEST INFORMATION** |
| **A. DEMOGRAPHIC CHARACTERISTICS. First, I’d like to ask you some quick questions about yourself.**   1. What is the name of your Sublocation/Community? __________________________   2b What is the name of your village? _____________________________    **First Name Middle Name Surname**   1. What is the name of your compound?   **First Name Middle Name Surname**   1. What is your name?   **Hour Minute**  **Consent Start time**: :     1. Consent complete? Yes  **Record pre-test start time**   No **Skip to Section C “MOBILE PHONE ASSESSMENT”**  **Hour Minute**  **Pre-test start time: : :**  **Day Month Year**   1. What is your date of birth? / **/** 2. How old are you? _______ (years) 3. What is your current relationship status? Single (no partner) Single (with partner) Married   Separated/Divorced Widowed   1. Do you currently live with your partner? Yes No 2. How many children have you given birth to? 3. How many children do you have under the age of 13? 4. What is the highest level of education you’ve completed? None/some primary Completed primary   Some secondary Completed secondary College and above   1. Are you currently working outside of the home? 2. Did you have to miss work to come to this campaign? 3. How did you get travel here today? Walked Paid for motorcycle Matatu   Own transportation (motorcycle, private vehicle) Other: _________________________   1. How much did it cost for you to get to the campaign today? 2. How many kilometers did you travel from your home to the campaign? 3. Did you come with anyone today? If so, who? Friend Relative Husband Children other: _______ 4. Did your partner, family member or anyone else important to you tell you to come? 5. Did your partner, family member or anyone else important to you tell you not to come?   **B. HEALTH AND SCREENING. Next, I have some questions about your health.**   1. Have you ever been screened for cervical cancer before? This might have been with a test called VIA, which is visual inspection with acetic acid. Yes No **Skip to Q23** Don’t know  **Skip to 22**   **Year**  21b. When were you last screened for cervical cancer? (Enter **9999** for ‘Don’t know’)  21c. Which type of screening was done? VIA/VILI Pap Smear HPV Don’t know  21d. Were any of those screening results abnormal? This could also be called a positive result.  Positive Negative Don’t know   1. Have you had any previous treatment for cervical cancer or precancer?   Yes No **Skip to Q23**  Don’t Know **Skip to Q23**  22b. Please describe previous treatment:  Cryotherapy Radiation Surgery  Chemotherapy LEEP Don’t Know Other specify________________   1. Have you ever been tested for HIV? Yes **Go to Q24** No **Skip to Q24f**   Don’t Know  **Skip to Q24f**  Refused **Skip to Q24f**   1. Do you remember the date when you were last tested? Yes **Go to Q24b** No **Go to Q24c**   **Day Month Year**  24b. Date of last HIV test: / /  **(*If participant can’t remember the specific date, have her provide just the year, or just the month & year)***  24c. What was the HIV test result? Positive **Go to Q24d** Negative **Go to Q24f**  Don’t know  **Skip to Q24f**  Refused  **Skip to Q24f**  24d. Are you in care? Yes **Go to Q24e** No **Go to Q24f**  24e. What is the name of the HIV treatment center? _______________________________  24f. Do you have any chronic medical conditions, or have you recently had other interactions with the healthcare system?  Yes **Go to Q24g** No **Go to Q25a**  24g. Please describe this other medical condition. Check all boxes that apply.  Headache Sore throat Breathing problems Dizziness/lightheadedness  Diarrhea Swollen lymph nodes Back/neck/leg pain Joint pain  Nausea and vomiting Rash Chest pain Eye/vision problems  Fatigue Fever Heart problems Vaginal pain  Aching muscles Abdominal pain Diabetes Other  List any other conditions that are not listed above: __________________________________________________  25a. How often do you feel depressed, down or hopeless? Almost every day Most days Some days Never  25b Have you ever been diagnosed with depression? Yes  No **Skip to Q26**  25c Did you seek help with depression? Yes No   1. Do you currently use FP/contraceptives? Yes **Go to Q26b** No **Go to Q27**   Not sexually active  **Skip to Q27** Refused **Skip to Q27** Don’t Know **Skip to Q27**  26b.Which type of FP/contraception are you currently using? (***select all mentioned***):  Male condom Diaphragm Female sterilization  Female condom Emergency contraceptive Vasectomy  Injectable/Depo Abstinence Implant  Birth control pills IUCD Patch  Other, specify: ______________________   1. Are you currently pregnant? Yes  No Don’t know   **C. MOBILE PHONE ASSESSMENT. Now, I’d like to ask you a few questions about your use of mobile phones.**   | C1. Do you ever use a mobile phone? | Yes  No (**skip to C6**)  Refused  Don’t Know | | | --- | --- | --- | | C2. Whose mobile phone do you regularly use?  **(Don’t read answer choices. Check all that apply.)** | My own  Spouse/partner  Child  Other family  Friend  Neighbor  No one  Other  Refused  Don’t Know |  | | C3. How many days per week do you typically use a mobile phone?  (**The participant may estimate. Please enter 0 if No contact number/no phone use, 88 if Refused, 99 if Don’t know)** | 0  1  2  3  4  5  6  7  88 - Refused  99 - Don’t Know |  | | C4. In the past year, how many different cell phone numbers (or SIM cards) have you used as your primary contact number?  (**The participant may estimate. Please enter 0 if No contact number/no phone use, 88 if Refused, 99 if Don’t know**) | 0 - Don’t have a contact number / no phone use  88 - Refused  99 - Don’t know |  | | C5. Please think about the mobile phone you use most frequently. How often are you not able to use the phone because of a problem? For example, if the phone is out of credit or the network is not working.  **(Read the answer choices aloud.)** | At least once a day  At least once a week  At least once a month  Other (less than once a month)  Never (it is always working)  Refused  Don’t Know |  | | C6. How comfortable are you receiving and reading SMS messages by phone?  **(Read answer scale aloud.)** | Very comfortable  Comfortable  Not comfortable  Very uncomfortable  Not able to  Refused  Don’t Know |  | | C7. How comfortable are you writing and sending SMS text messages by phone?  (Read answer scale only as necessary. Participant should answer about her own comfort, regardless of phone access.) | Very comfortable  Comfortable  Not comfortable  Very uncomfortable  Not able to  Refused  Don’t Know |  | | C8. How comfortable would you be with receiving your confidential and sensitive information, such as your cervical cancer screening result, by SMS message?  **(Read answer scale only as necessary.)** | Very comfortable  Comfortable  Uncomfortable  Very uncomfortable  Refused  Don’t Know |  |   If consent **complete**, continue to **Q28.**  If consent **not complete**, skip to **Q33.** |
| **SPECIMEN COLLECTION**  **Hours Minute**  Specimen collection start time (time kit given to client): **:**        **** Scan barcode on the brochure, pick one barcode from the brochure and attach it on the sample collection kit and hand the kit plus the brochure with the two remaining barcodes to the participant for sample collection. Send them for self-collection directing them to the Post-test desk when done with specimen collection.** |

| **PARTICIPANT POST-TEST INFORMATION** |
| --- |
| **Hour Minute**  **Post-test start time: :**  If testing is complete **(if the first test was not successful give participant another kit for a second attempt)**, pick a barcode from the brochure and attach it on the sample tracking Log. Scan and leave the remaining barcode on the brochure. Instruct participant to keep the barcode on the brochure safe as this will be used as the clinic ID when/if they go for treatment   1. Were you able to complete testing? Yes Scan Barcode **Go to Q29** No **Skip to Q39** 2. How hard was it to understand the self-test instructions? Circle one.   Very hard Somewhat hard Neutral Somewhat easy Very easy Refused Don’t know   1. Was privacy adequate? Circle one.   Yes No Refused Don’t know     1. How much discomfort, if any, did you experience when you used the self-test? Circle one.   No discomfort Little discomfort Neutral Some discomfort A lot of discomfort Refused Don’t know   1. How much physical pain, if any, did you have when you used the self-test? Circle one.   No pain Little pain Neutral Some pain A lot of pain Refused Don’t know     1. Do you have access to a phone? Yes No **Go to Q34**   33b. Participant phone number:  **Alternative contact if available:**   1. If your results are normal, how would you prefer to receive your test results?   SMS Phone call Home visit **(if no access to phone)**     1. If your results show that you have HPV, how would you prefer to receive your test results?   SMS **Go to 36** Phone call **Skip to 37** Home visit from CHW **Skip to 37**     1. Which language do you prefer for text messages? **(ask if SMS is the preferred notification option)**   English Dholuo  Kiswahili Other, specify _________________________________   1. Could you provide directions in case we need to find you in the future? ___________________________ ___________________________________________________________________________________  | 1. Women who test positive for HPV need follow-up care and possibly treatment at a nearby facility. If you have a positive test result, will you try to get follow-up care and treatment? Would you say you…(***Read answer choices aloud***) | Definitely won't Probably won't  Probably will  Definitely will  Don’t Know  **🡪 ALL Go to Q40** | | --- | --- |   **Fill only if testing was not completed**   1. Why were you unable to complete testing today? (***Do not read aloud. Mark all that apply***)   Menses Not enough time to wait  Did not understand instructions Inadequate supplies/equipment at site  Did not have adequate privacy No trained staff member  Too uncomfortable/painful Changed my mind  Fear of procedure Pregnant  Seems too complicated I’ve had a hysterectomy  I wouldn’t have HPV Other, specify  __________________________________________________________     1. Would you be willing to use the HPV self-test in the future? Yes No 2. Would you recommend the HPV self-test to a friend? Yes No 3. Do you have any remaining questions about testing? Yes Go to **Q42b** No   42b. Please add any additional comments or questions here:_______________________________      **Hours Minutes**  **Post-test stop time: :** |
